# Aging trajectories of subscales in higher-level functional capacity among community-dwelling older Japanese adults: the Otassha study

**DOI:** 10.1007/s40520-024-02791-x

**Published:** 2024-06-21

**Authors:** Hisashi Kawai, Keigo Imamura, Manami Ejiri, Yoshinori Fujiwara, Kazushige Ihara, Hirohiko Hirano, Hiroyuki Sasai, Shuichi Obuchi

**Affiliations:** 1 Tokyo Metropolitan Institute for Geriatrics and Gerontology, 35-2 Sakae-cho, Itabashi-ku, Tokyo, 173-0015 Japan; 2 Faculty of Medicine, Hirosaki University, Aomori, Japan

**Keywords:** Higher-level functional capacity, Tokyo Metropolitan Institute of Gerontology Index of Competence, Subscale, Group-based trajectory modeling

## Abstract

**Background:**

Maintaining higher-level functional capacity is important for independent living in older age. The aging trajectory of the Tokyo Metropolitan Institute of Gerontology Index of Competence (TMIG-IC) has three patterns; however, the subscale patterns are unclear.

**Aims:**

This study aimed to clarify the aging trajectory patterns of the TMIG-IC subscales among community-dwelling older Japanese.

**Methods:**

Participants were 3,169 community-dwelling older Japanese who participated in the 2012–2022 mail survey of the Otassha study. The aging trajectory patterns of the TMIG-IC total and subscale scores for those aged 65–90 years were identified using group-based trajectory modeling. Further, the combination frequency of the subscale trajectory patterns was determined.

**Results:**

Three patterns were identified: early-onset decreasing, late-onset decreasing, and high-stable.

**Discussion:**

The instrumental activities of daily living (IADL) trajectory was maintained until approximately 80 years of age; however, chronic disease prevailed the most in the early-onset decreasing pattern. The early-onset decreasing pattern of intellectual activity (IA) was present in 25% of participants, showing impaired IA from 65 years of age. The late-onset decreasing pattern of social roles (SR) was present in 30% of participants, showing a sharp decline compared to other subscales. For many people, the patterns of decrease in SR and IA overlapped.

**Conclusions:**

To maintain higher-level functional capacity, interventions that include disease management and prevention of decline in IADL and increase the awareness of the social support provided throughout old age and interventions for people with an early decline in IA should be implemented.

**Supplementary Information:**

The online version contains supplementary material available at10.1007/s40520-024-02791-x.

## Introduction

Higher-level functional capacity comprises the top three levels, as defined by Lawton [1]: social role (SR), intellectual activity (IA), and instrumental activities of daily living (IADL). Higher-level functional capacity is associated with adverse health outcomes such as death [2, 3], stroke [4], dynapenia [5], and subjective memory complaints [6]. Maintaining a higher functional capacity to live independently in old age is extremely important.

The 13-item Tokyo Metropolitan Institute of Gerontology Index of Competence (TMIG-IC) is a questionnaire developed to evaluate higher-level functional capacity [7] (Supplementary Table 1). As TMIG-IC can evaluate the subscales of SR, IA, and IADL, it has been used in several previous studies to analyze age-related changes in its total and subscale scores [8, 9], to determine the effects of declines in subscales on stroke and death [2, 4], and to examine health behaviors that are predictors of the decline of total and subscale scores [10]. These studies show how higher-level functional capacity declines with age, how the decline affects adverse health outcomes, and what the risk factors in the decline are.

The TMIG-IC was developed approximately 30 years ago, and high-quality longitudinal data have been used to determine its aging trajectory patterns [3, 11]. Analyzing aging trajectory patterns can clarify what patterns exist in the decline of higher-level functional capacity in older adults and help determine the target proportion and timing of interventions for maintaining higher-level functional capacity [12].

Taniguchi et al. [3] identified the aging trajectory patterns of TMIG-IC using group-based trajectory modeling from longitudinal data collected from 2001 to 2011 in a cohort of community-dwelling older adults. The patterns were “high-stable,” in which high function is maintained until later in life; and “late-onset decreasing,” in which high function is maintained until approximately 80 years of age but gradually declines thereafter. Further, they include “early-onset decreasing,” in which the score gradually declines after 65 years; and “low-decreasing,” in which the score at 65 years is already low and declines linearly. The researchers found higher mortality and medical costs in the low-decreasing group.

Iwasaki and Yoshihara [11] identified three aging trajectory patterns, such as “high/stable,” “middle/decline,” and “low/decline” from the longitudinal data of the TMIG-IC collected from 1998 to 2008 in a cohort of community-dwelling older adults. They reported that poor dentition status is associated with a decline in higher-level functional capacity. These previous studies show aging trajectory patterns that suggest the number and age of people in the declining group who should be targeted for intervention, which will be useful for preventing declines in higher-level functional capacity. However, the baseline survey was conducted more than 20 years ago, subscale trajectory patterns were not identified, and sex differences in trajectory patterns were not examined.

A prior study using integrated cohort data of older Japanese adults found that walking speed, grip strength, and IADL scores had improved compared to data for the same age group 10 years ago. Further, they noted rejuvenation among older Japanese adults [13]. Thus, aging trajectory patterns should be clarified using newer data. Clarifying the trajectory patterns of subscales and determining whether there are sex differences could lead to intervention strategies for maintaining higher-level functional capacity. Thus, this study aimed to clarify the aging trajectory patterns of the TMIG-IC subscales and their sex differences.

## Materials and methods

### Participants

Participants were community-dwelling older Japanese people who participated in the 2012–2022 mail survey of the Otassha study 2011 cohort. This cohort selected all residents of nine areas in Itabashi Ward, Tokyo, Japan (excluding nursing home residents and other cohort study participants) and has been conducting a site-invited survey since 2011. A mail survey was conducted in 2012, and self-administered questionnaires were mailed to 7,015 people in the baseline mail survey in October 2012, of whom 3,696 responded (response rate: 52.7%). Follow-up mail surveys were conducted in October 2013, 2014, 2017, 2018, 2019, 2020, 2021, and 2022. The mail survey for this cohort was published elsewhere [2, 14, 15]. The mail survey for this cohort includes a space to indicate whether the person is completing the survey independently or if a family member completed it for them. In the baseline survey, we confirmed that 93.7% of the responses were completed by the participant, and 3.3% were completed by someone else writing the participant’s answers.

**Fig. 1:**
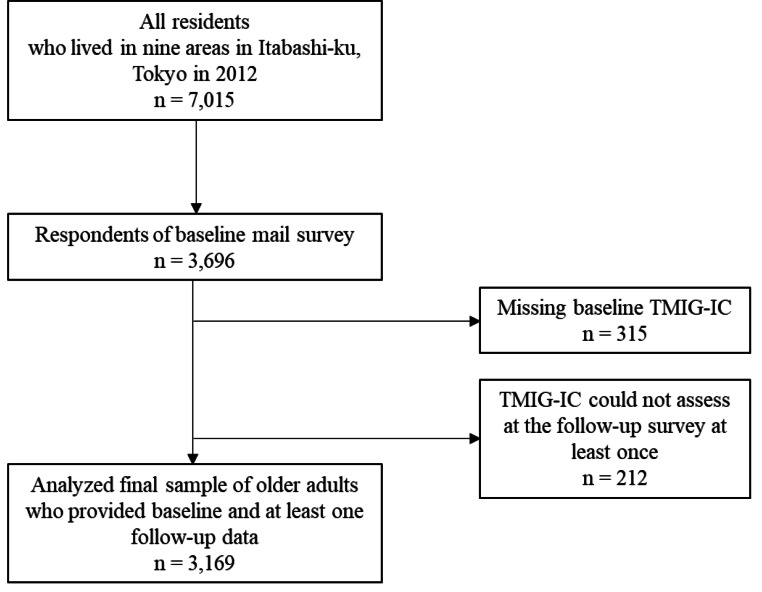
Participant flowchart TMIG: Tokyo Metropolitan Institute of Gerontology Index of Competence.

Participants were 3,169 older adults who responded to the TMIG-IC in the 2012 baseline mail survey and at least one follow-up survey (Fig. 1). The average number of surveys (SD) was 5.8 (2.5) and the number of observations was 17,908.

Participants provided written informed consent to use their data for this study. This study was approved by the Ethics Committee of the Tokyo Metropolitan Institute of Gerontology (no. R21-033). This study was conducted in accordance with the guidelines of the World Medical Association Declaration of Helsinki.

### Higher-level functional capacity

Higher-level functional capacity was evaluated using the TMIG-IC. This index comprises 13 items and can evaluate the subscale scores of IADL (items 1–5), IA (items 6–9), and SR (items 10–13) [2, 7].

If the response was “Yes,” 1 point was added; if “No,” 0 points were added. The total score ranges from 0 to 13 points, with higher scores indicating higher-level functional capacity. Regarding subscale scores, IADL had a range of 0–5 points, IA and SR had a range of 0–4 points, and a less-than-perfect score indicated impairment in the functioning of each subscale [2, 8, 11].

### Other variables

Age, sex, chronic diseases, living alone status, self-rated health, perceived financial status, and social participation were examined in the baseline survey. The presence or absence of each chronic disease (i.e., hypertension, diabetes mellitus, stroke, cancer, and heart disease) was recorded. The questions regarding self-rated health provided four choices: *very healthy*, *sufficiently healthy*, *not very healthy*, and *not healthy*. Perceived financial status was evaluated using five options: *very comfortable*, *slightly comfortable*, *neither comfortable nor hard*, *slightly hard*, and *very hard*. Social participation was examined in several activity groups, including neighborhood associations, senior citizen clubs, hobby groups, sports groups, and volunteer groups [15].

### Statistical analysis

The aging trajectory patterns of the total and subscale scores of the TMIG-IC for all participants, men, and women aged 65–90 years were identified using group-based trajectory modeling [16] from the longitudinal data of 2012–2020. Group-based trajectory modeling was conducted using a cnorm model of the TRAJ in STATA 17.0 (StataCorp LLC, College Station, Texas, United States). Three and four groups of total TMIG-IC scores were investigated using a quadratic curve model, and we identified four trajectory patterns similar to those reported in a previous study [3]. However, because the low-decreasing group comprised a small proportion (less than 10%), we accepted three trajectory patterns for each subscale from the perspective of the Bayesian information criterion value. The cutoff for TMIG-IC, which indicates difficulty in living independently, is reported as nine points or less [17]. It is reasonable that the lowest TMIG-IC trajectory pattern starts around nine points from the perspective of maintaining higher-level functional capacity for independent living.

The differences in all variables between the trajectory pattern groups of the TMIG-IC total score were examined using the Z test for categorical variables, and one-way analysis of variance and Bonferroni’s post-hoc test were employed for continuous variables. The frequency distribution of the trajectory group combinations (27 ways) for each subscale score was examined. The distribution of the subscale trajectory group combinations in each TMIG-IC trajectory pattern was also examined. SPSS version 27.0 (IBM Japan, Ltd., Tokyo, Japan) was used for statistical analyses other than group-based trajectory modeling.

## Results

**Fig. 2:**
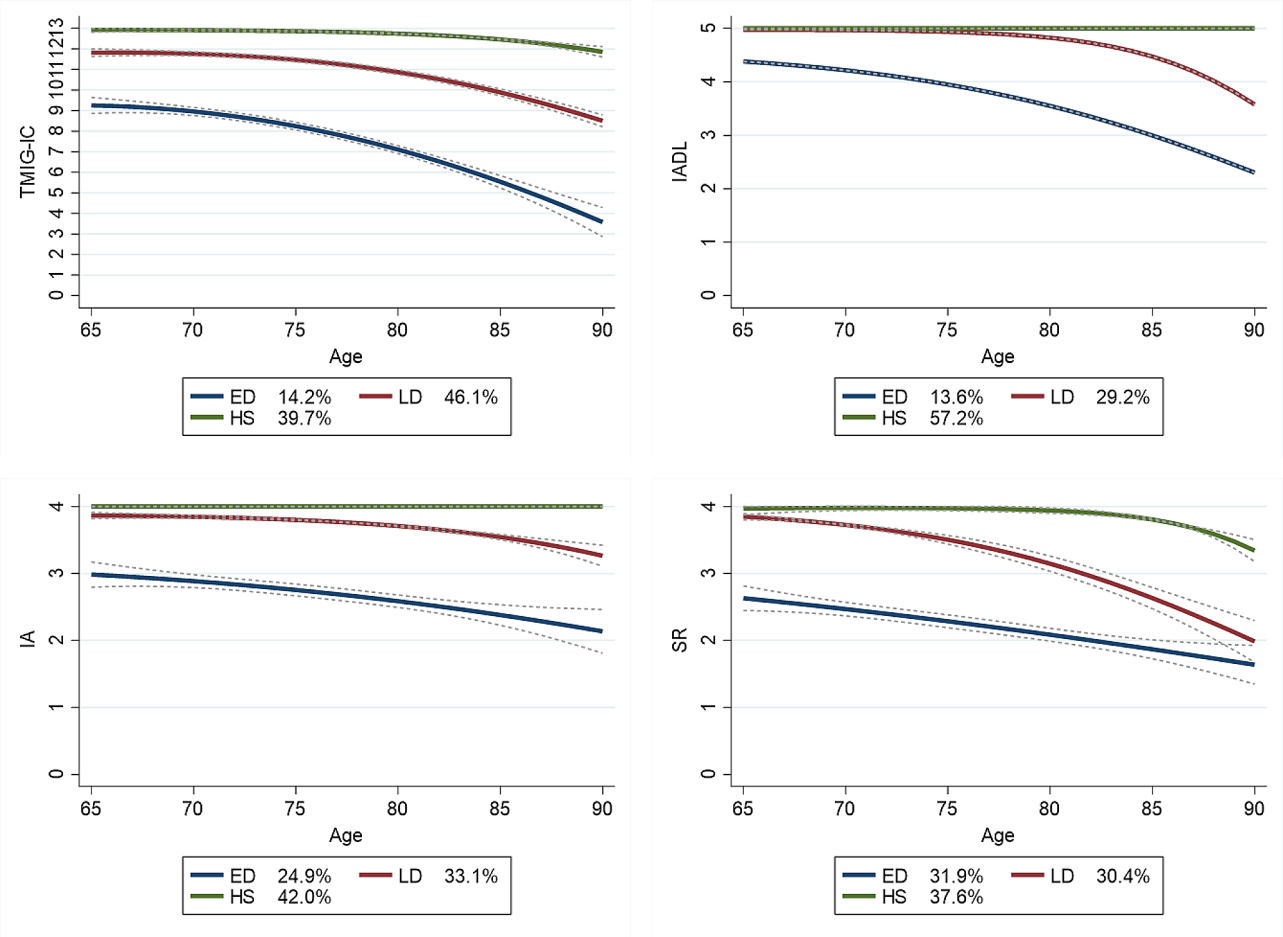
Aging trajectories of the TMIG-IC and its subscales among community-dwelling older adults The legends show the percentage of participants included in each trajectory. Dotted line: 95% confidence interval, TMIG-IC: Tokyo Metropolitan Institute of Gerontology Index of Competence, IADL: instrumental activities of daily living; IA: intellectual activity; SR: social role; ED: early-onset decreasing, LD: late-onset decreasing, HS: high-stable

Three aging trajectory patterns of the TMIG-IC total score were identified: high-stable (39.7%), late-onset decreasing (46.1%), and early-onset decreasing (14.2%; Fig. 2, upper left). The high-stable group maintained a perfect score of 13 points until approximately 90 years of age. The late-onset decreasing group maintained 12 points until 75 years of age but gradually declined thereafter. The early-onset decreasing group was already approximately nine points at the age of 65 years, followed by a rapid decline.

The aging trajectory patterns of the IADL score were identified as high-stable (57.2%), late-onset decreasing (29.2%), and early-onset decreasing (13.6%; Fig. 2, upper right). The high-stable group maintained a perfect score of five points. The late-onset decreasing group maintained 5 points until 75 years of age, but declined thereafter. The early-onset decreasing group scored approximately four points at the age of 65 years, followed by a rapid decline.

The aging trajectory patterns of the IA score were identified as high-stable (42.0%), late-onset decreasing (33.1%), and early-onset decreasing (24.9%; Fig. 2, lower left). The high-stable group maintained a perfect score of four points. The late-onset decreasing group scored approximately four points at 65 years but declined slightly after 70 years. The early-onset decreasing group scored approximately three points at the age of 65 years, followed by a rapid decline.

The aging trajectory patterns of the SR score were identified: high-stable (37.6%), late-onset decreasing (30.4%), and early-onset decreasing (31.9%; Fig. 2, lower right). The high-stable group maintained a perfect score of four points until 80 years, which declined slightly thereafter. The late-onset decreasing group scored approximately four points at 65 years of age, followed by a rapid decline. The early-onset decreasing group scored less than three points at the age of 65 years, which declined with age thereafter. Although no significant differences in these trajectory patterns were observed between men and women, the proportion of male participants with late-onset and early-onset decreasing patterns was higher than that of women (Supplementary Figs. 1 and 2).

Regarding the baseline characteristics, the proportion of women was significantly higher in the high-stable group than in the other pattern groups (Table 1). The prevalence of diabetes and stroke was significantly higher in the early-onset decreasing pattern group than in the other pattern groups, and the prevalence of heart disease was significantly lower in the high-stable pattern group than in the other pattern groups. Living alone, non-participation in social groups, poor self-rated health, and poor perceived financial status were higher in the early-onset and late-onset decreasing groups than in the high-stable group. Age was significantly higher in the late-onset decreasing group than in the early-onset decreasing and high-stable groups. The total score and each subscale score of the TMIG-IC were highest in the order of early-onset decreasing, late-onset decreasing, and high-stable.

**Table 1:** Baseline characteristics of the trajectory groups

		TMIG-IC trajectory groups					
		(a) Early-onset decreasing		(b) Late-onset decreasing		(c) High-stable	
		n	%	n	%	n	%
Sex	Female	175	40.6	784	52.5^a^	794	63.8^a, b^
Chronic disease	Hypertension	186	45.5	592	41.8	460	38.9
	Diabetes mellitus	83	20.3^b, c^	185	13.1	125	10.6
	Stroke	40	9.8^b, c^	57	4.0	33	2.8
	Cancer	22	5.4	66	4.7	41	3.5
	Heart disease	68	16.6^c^	200	14.1^c^	116	9.8
Living alone		125	29.3^b, c^	336	22.6	234	19.0
No group participation		225	57.1^b, c^	572	40.1^c^	246	20.4
Self-rated health	Very healthy	14	3.4	128	9.0^a^	193	16.1^a, b^
	Healthy enough	194	47.8	977	68.6^a^	882	73.7^a, b^
	Not very healthy	117	28.8^b, c^	259	18.2^c^	111	9.3
	Not healthy	81	20.0^b, c^	60	4.2^c^	11	0.9
Perceived financial status	Very comfortable	5	1.2	50	3.4	40	3.3
	A little comfortable	68	16.1	458	30.9^a^	477	38.8^a, b^
	Neither comfortable nor hard	177	41.9	630	42.5	536	43.6
	A little hard	116	27.5^b, c^	282	19.0^c^	153	12.4
	Very hard	56	13.3^b, c^	62	4.2^c^	23	1.9
		Mean	SD	Mean	SD	Mean	SD
Age (years)		72.3	5.4	73.2^a, c^	5.6	72.4	5.2
TMIG-IC total score		8.5	2.6	11.5^a^	1.4	12.8^a, b^	0.5
IADL score		4.0	1.5	4.8^a^	0.5	5.0^a, b^	0.1
IA score		2.7	1.1	3.6^a^	0.6	4.0^a, b^	0.2
SR score		1.8	1.2	3.0^a^	1.0	3.9^a, b^	0.3

Superscripts indicate significantly greater value than that of the trajectory groups shown by the letter types (*p* < .05)

TMIG-IC: Tokyo Metropolitan Institute of Gerontology Index of Competence; IADL: instrumental activities of daily living; IA: intellectual activity; SR: social role

Regarding the subscale trajectory pattern group combinations, all high-stable patterns were most common with a prevalence of 25.0%, followed by IADL high-stable, IA high-stable, and SR late-onset decreasing with a prevalence of 10.2%; IADL high-stable, IA late-onset decreasing, and SR high-stable with a prevalence of 6.8%; IADL and IA high-stable and SR early-onset decreasing with a prevalence of 6.5%; and IADL high-stable, IA, and SR early-onset decreasing with a prevalence of 6.0% (Table 2).

**Table 2:** Combined trajectory groups of the TMIG-IC subscales

	Trajectory groups of subscales			n	%
	IADL	Intellectual activity	Social role		
1	HS	HS	HS	793	25.0
2	HS	HS	LD	323	10.2
3	HS	LD	HS	215	6.8
4	HS	HS	ED	206	6.5
5	HS	ED	ED	190	6.0
6	HS	LD	LD	176	5.6
7	ED	ED	ED	169	5.3
8	HS	LD	ED	163	5.1
9	HS	ED	LD	114	3.6
10	LD	LD	LD	100	3.2
11	HS	ED	HS	87	2.7
12	LD	ED	ED	72	2.3
13	ED	LD	ED	68	2.1
14	LD	LD	HS	60	1.9
15	LD	HS	HS	58	1.8
16	LD	LD	ED	55	1.7
17	LD	HS	LD	55	1.7
18	LD	ED	LD	46	1.5
19	ED	ED	LD	42	1.3
20	ED	HS	ED	36	1.1
21	ED	LD	LD	31	1.0
22	ED	ED	HS	26	0.8
23	LD	HS	ED	26	0.8
24	ED	HS	LD	17	0.5
25	ED	HS	HS	15	0.5
26	LD	ED	HS	14	0.4
27	ED	LD	HS	12	0.4
			Total	3169	100.0

TMIG: Tokyo Metropolitan Institute of Gerontology Index of Competence

ED: early-onset decreasing, LD: late-onset decreasing, HS: high-stable

**Fig. 3:**
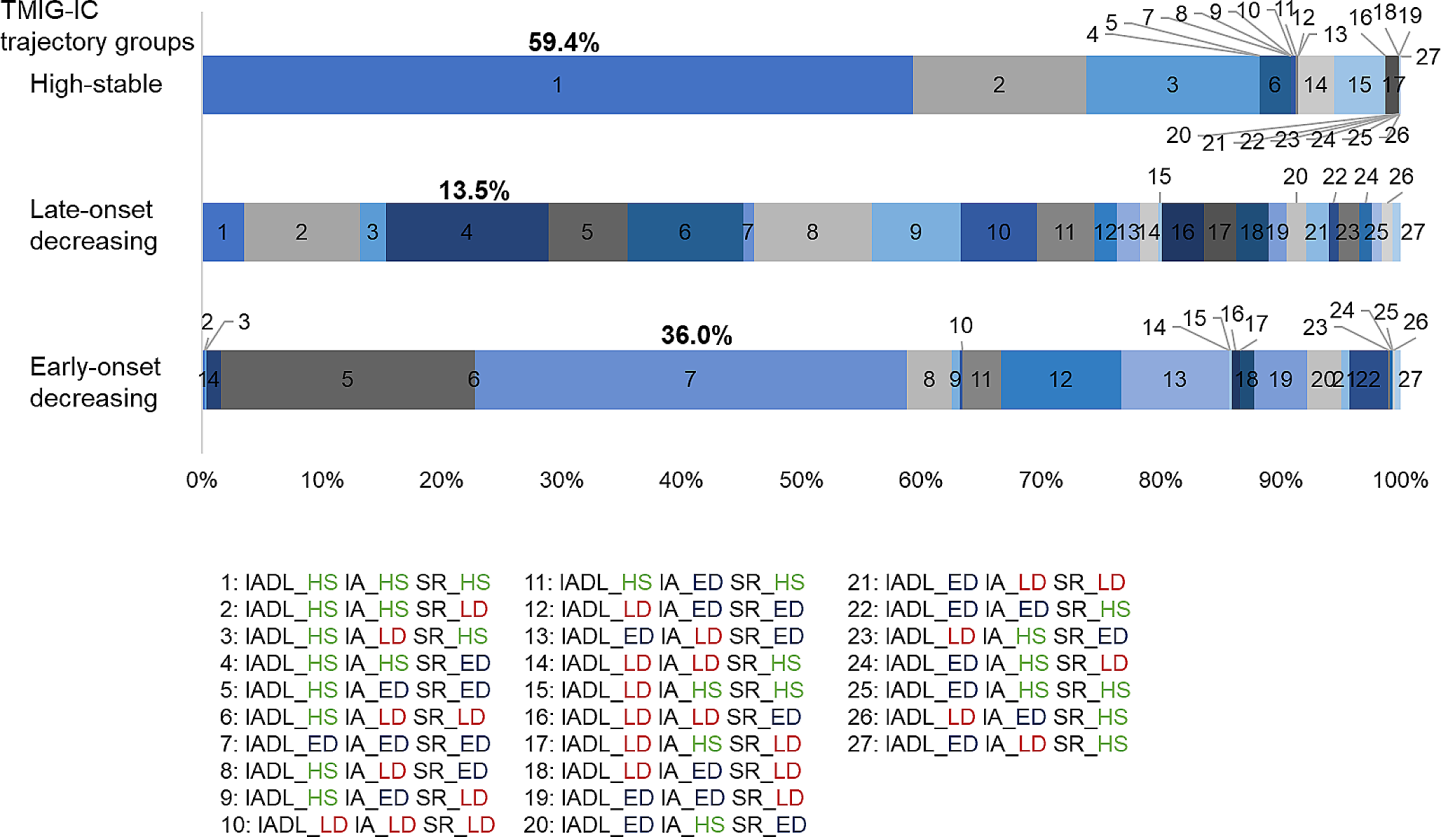
Combination of aging trajectory patterns of TMIG-IC and each subscale

The most common combinations of the subscale trajectory pattern groups in the TMIG-IC high-stable, late-onset decreasing, and early-onset decreasing groups were all high-stable patterns, IADL and IA high-stable, SR early-onset decreasing, and all early-onset decreasing, respectively (Fig. 3).

The percentages of most common combinations in the each TMIG-IC trajectory group were displayed in bold. TMIG: Tokyo Metropolitan Institute of Gerontology Index of Competence; IADL: instrumental activities of daily living; IA: intellectual activity; SR: social role; ED: early-onset decreasing, LD: late-onset decreasing, HS: high-stable.

## Discussion

We identified the aging trajectory patterns of the TMIG-IC based on approximately 18,000 observation data points from approximately 3,000 older adults over 10 years from 2012 to 2022. A previous study [3] showed the aging trajectory patterns of the TMIG-IC total score in community-dwelling older Japanese individuals: high-stable, late-onset decreasing, early-onset decreasing, and low-decreasing groups. As we combined the early-onset decreasing and low-decreasing groups, our study identified three groups that were very similar to those of a previous study [3]. The patterns presented in this study represent the aging trajectory of TMIG-IC among older Japanese individuals. The data in this study were 10 years newer than those in the previous study. The TMIG-IC total scores at baseline were 12.8 for high-stable, 11.5 for late-onset decreasing, and 8.5 for early-onset decreasing, whereas they were 12.8, 11.6, 9.8, and 6.4, respectively, in the previous study, which were similar. A prior study reported that the several functions of older Japanese had improved compared to 10 years ago [13]. Although higher-level functional capacity was also improved, the improvement may not have been captured owing to the ceiling effect of TMIG-IC. A significantly higher-level functional capacity index has been developed from the perspective that the health status of older Japanese people is improving [18]. In the future, it will be necessary to clarify the aging trajectory patterns of this new index.

For the subscale trajectory patterns of higher-level functional capacity, some studies have reported the trajectory patterns of IADL and basic ADL (BADL) [19, 20]; however, to our knowledge, no study has identified the trajectory patterns of IA and SR. This study found that the trajectory pattern groups of IADL were 57.2% for high-stable, 29.2% for late-onset decreasing, and 13.6% for early-onset decreasing. Further, it showed that the proportion for high-stable patterns was more and that for the early-onset decreasing group was less than those in a previous study for the trajectory patterns of IADL among Chinese older adults [20]. Although it is difficult to compare simply because the indicators used for IADL assessment differed between the two studies, the previous study identified three groups: a low-risk group in which the risk remained low, an increasing risk group in which the risk increased gradually, and a high-risk group in which high risk had continued since baseline. The low- and high-risk groups in the previous study could be compared with the high-stable and early-onset groups in our study, respectively. The current results may suggest that many older Japanese maintain IADL. Even in the late-onset decreasing group, the IADL score was maintained until approximately 80 years of age. In Japan, local governments have been implementing preventive care and frailty program such as exercise and nutrition interventions for older people with risks of IADL impairment and frailty [21, 22]. The results concerning IADL in the late-onset decreasing group could be owing to the effects of care and frailty prevention programs. In the early-onset decreasing group, the prevalence of diabetes and stroke at baseline was high, which is consistent with previous studies showing that diseases lead to early-onset decreasing in BADL and higher-level functional capacity [3, 19]. Another study showed that the number of physical functioning difficulties increased with age as the number of diseases increased [23]. Disease management and interventions are necessary to prevent IADL decline in parallel with diseases in early old age. Specific intervention strategies include providing regular health checkups and health education [21].

In the IA trajectory pattern, compared with the IADL trajectory pattern, fewer people were included in the high-stable group, whereas slightly more participants were included in the late-onset decreasing and early-onset decreasing groups. The early-onset decreasing group comprised 25% of all participants who already had a 1-point decline in IA score; that is, they had IA impairment at 65-years-old. Exposure to IAs is associated with subsequent improvements in IADL among older people [24], and even in patients with depressive symptoms who are at a high risk of care needs, increased learning activities are associated with less future care needs [25]. To maintain higher-level functional capacity, early intervention is important for patients in the IA early-onset decreasing group. Possible interventions to increase IA include participation in intellectual activities [24] and health education to increase interest in health.

For SR, those in the early-onset decreasing group were even greater than those in the IA group, suggesting the need for earlier intervention. The slope of the late-onset decreasing pattern, which included approximately 30% of all participants, was rapid, indicating the existence of individuals whose SR declined rapidly with age. A previous study reported that social function declines more slowly than physical function [26]. Our previous study suggested that social interaction is maintained in old age [15]. However, regarding the pattern of the rapid decline in SR, the SR question on the TMIG-IC could have included awareness of the social support provided, such as being called on for advice, visiting sick friends, and initiating conversations with young people. High levels of social function slow physical function decline [27], and interventions that increase awareness of the social support provided—that is, the recognition of being helpful to others—are necessary throughout old age. For example, it is possible to provide opportunities for social engagement activities through intergenerational exchange [28].

To date, no study has reported sex differences in the aging trajectory patterns of the total and subscale scores of the TMIG-IC. A previous study reported that physical function trajectories were similar between men and women among community-dwelling older adults; however, women exhibited a later but faster decline compared to men [29]. One study suggested that the physical function of stroke patients was lower in women than in men; however, there was no difference in the declining trend [30]. A study of community-dwelling older Japanese individuals reported that the aging trajectories of walking speed and grip strength were parallel [31]. This study found that the trajectories of higher-level functional capacity and its subscales were similar between men and women, with no significant differences. However, the proportion of late-onset and early-onset decreasing patterns was higher in men than in women, and women maintained higher-level functional capacity. Therefore, the aforementioned interventions to increase IA and SR are particularly important in men.

For all subscale trajectory combinations, high-stable was the most common with a prevalence of 25.0%, followed by late-onset decreasing in only SR (10.2%) and late-onset decreasing in only IA (6.8%). The TMIG-IC late- and early-onset decreasing patterns included combinations with decreasing SR, suggesting the importance of intervention in SR. In the baseline survey conducted in the same cohort as this study, the most common combination of TMIG-IC impairment was none (47.1%), followed by SR only (21.8%), and SR and IADL impairments (6.8%) [2]. Compared with participants who continued to participate in the longitudinal study, 22.1% experienced a decline in one of the subscales during the follow-up period, and 10.2% experienced a late decline only in SR. As many participants with late-onset and early-onset decreasing patterns in SR also had late-onset and early-onset decreasing patterns in IA, they should be major targets for intervention. Thus, examining the frequency of trajectory pattern combinations of subscales, as well as baseline impairment combinations, can inform specific intervention strategies. As a previous study reported combinations of overlapping frailty [32], future research is needed on combinations of these trajectory patterns.

### Limitations

As this study was conducted in an urban area of Japan, the generalizability of the results is limited. Regional differences in the prevalence of subscale decline in higher-level functional capacity have been reported [9]. Therefore, future studies that consider other regions are warranted. Of the 3,381 patients for whom baseline TMIG-IC data could be obtained, 212 dropped out of follow-up (dropout rate 6.3%). The effect of attrition bias is not to be large enough to alter the estimated trajectories. However, the response rate for the mail survey in this study was approximately 50%, and there is a selection bias. As this study used data from mail surveys with limited items, other potential covariates such as body size, alcohol consumption, smoking habits, education, and cognitive function could not be considered. In particular, a major limitation of this study is that it does not address the possibility of mild cognitive impairment or cognitive impairment. However, since 88.1% of participants had no IADL impairment at baseline (data not shown), the data would include many people who are not significantly affected by cognitive decline.

A multifaceted examination of the factors that influence the trajectory patterns of the TMIG-IC subscales will help develop specific intervention strategies for maintaining higher-level functional capacity.

## Conclusions

This study examined the aging trajectory of the TMIG-IC subscales among community-dwelling older Japanese individuals and identified the following strategies for maintaining higher-level functional capacity. Interventions for disease management and prevention of a decline in IADL at an early age are necessary. Interventions for people with an early decline in IA and increasing awareness of the social support provided throughout old age are necessary. Men and those with overlapping patterns of decrease in SR and IA should be the major targets for intervention.

### Electronic supplementary material

Below is the link to the electronic supplementary material.

Supplementary Material 1

## Data Availability

No datasets were generated or analysed during the current study.
