# Supplementary material for: Aging trajectories of subscales in higher-level functional capacity among community-dwelling older Japanese adults: the Otassha study
Source: Aging Clin Exp Res. 2024 Jun 21;36(1):137. doi: 10.1007/s40520-024-02791-x (PMC11192685; doi:10.1007/s40520-024-02791-x)
Supplement: Supplementary file 1 — Supplementary Material 1 [file 40520_2024_2791_MOESM1_ESM.docx]

**Supplementary Table 1.** Each item that comprises subscales of the Tokyo Metropolitan Institute of Gerontology Index of Competence (TMIG-IC).

| Subscales | Items |
| --- | --- |
| Instrumental activities of daily living | 1. Can you use public transportation (bus or train) by yourself? |
|  | 2. Are you able to shop for daily necessities? |
|  | 3. Are you able to prepare meals by yourself? |
|  | 4. Are you able to pay bills? |
|  | 5. Can you handle your own banking? |
| Intellectual activity | 6. Are you able to fill out forms for your pension? |
|  | 7. Do you read newspapers? |
|  | 8. Do you read books or magazines? |
|  | 9. Are you interested in news stories or programs dealing with health? |
| Social role | 10. Do you visit the homes of friends? |
|  | 11. Are you sometimes called on for advice? |
|  | 12. Are you able to visit sick friends? |
|  | 13. Do you sometimes initiate conversations with young people? |

**
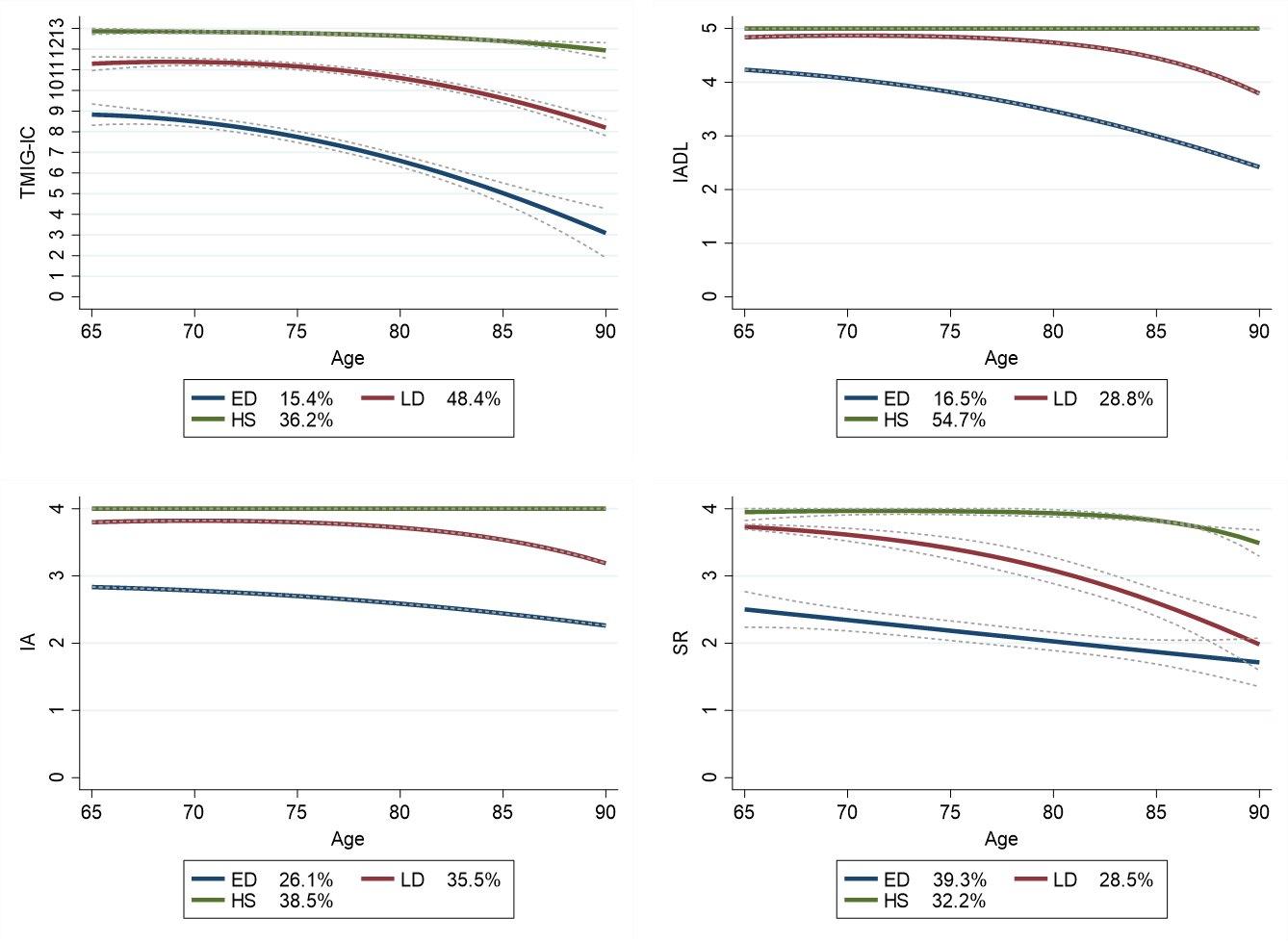
**

**Supplementary Fig. 1** Aging trajectories of the TMIG-IC and its subscales among men

The legends show the percentage of participants included in each trajectory. Dotted line: 95% confidence interval, TMIG: Tokyo Metropolitan Institute of Gerontology Index of Competence, IADL: instrumental activities of daily living; IA: intellectual activity; SR: social role; ED: early onset decreasing, LD: late-onset decreasing, HS: high-stable.


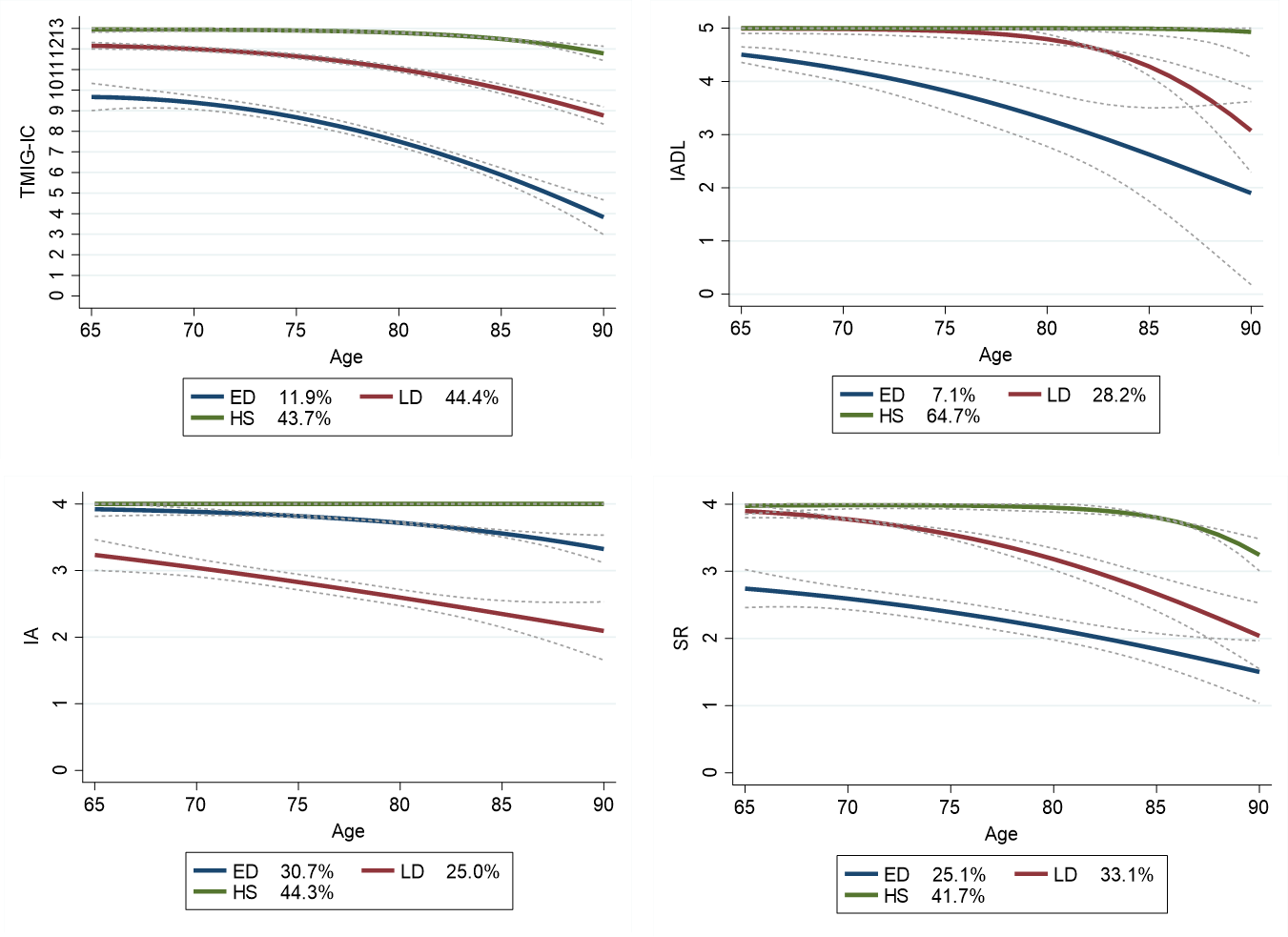


**Supplementary Fig. 2** Aging trajectories for the TMIG-IC and its subscales among women

The legends show the percentage of participants included in each trajectory. Dotted line: 95% confidence interval, TMIG: Tokyo Metropolitan Institute of Gerontology Index of Competence, IADL: instrumental activities of daily living; IA: intellectual activity; SR: social role; ED: early onset decreasing, LD: late-onset decreasing, HS: high-stable.
